# The role of *Veillonella* species in oral carcinogenesis: is prevalence linked to oral squamous cell carcinoma?

**DOI:** 10.3389/froh.2026.1740043

**Published:** 2026-05-08

**Authors:** Citra Fragrantia Theodorea, Nadhira Aurynna Azzahra, Erik Idrus, Fahrul Nurkolis, Ariadna Adisattya Djais, Izumi Mashima

**Affiliations:** 1 Department of Oral Biology, Faculty of Dentistry, Universitas Indonesia, Depok, Indonesia; 2 Institute for Research and Community Service, State Islamic University of Sunan Kalijaga (UIN Sunan Kalijaga), Yogyakarta, Indonesia; 3 Department of Oral Medical Science, School of Dentistry, Ohu University, Koriyama, Japan

**Keywords:** biomarker, carcinogenesis, inflammation, oral dysbiosis, oral microbiome, oral squamous cell carcinoma (OSCC), therapeutic target, *Veillonella* species

## Abstract

Oral squamous cell carcinoma (OSCC) remains a significant health challenge because of its aggressive nature and poor survival outcomes. While established risk factors such as tobacco use, alcohol consumption, and human papillomavirus play critical roles, increasing evidence suggests that oral microbial dysbiosis may contribute to carcinogenesis. Among oral commensals, *Veillonella* species have gained attention because of their ecological role in oral biofilms and metabolic interactions with other microbes, and have also been increasingly identified in altered abundances within OSCC patient samples. This narrative review synthesizes available clinical, epidemiological, and molecular studies investigating the prevalence and biological roles of *Veillonella* species in OSCC. Relevant English-language publications between 2000 and 2025 were identified through database searches in Pubmed Scopus and Web of Science using keywords related to *Veillonella*, oral microbiome, dysbiosis, and OSCC. The reviewed evidence reveals a dynamic and stage-dependent shift in *Veillonella* abundance during oral carcinogenesis. Several studies report enrichment of *Veillonella* in oral potentially malignant disorders and early tumorigenesis, whereas reduced levels are frequently observed in advanced OSCC. These findings suggest that *Veillonella* may function as an ecological modulator of tumor-associated microbiota rather than as a single pathogenic driver. Proposed mechanisms include metabolic cross-feeding with lactic-acid-producing bacteria, modulation of inflammatory pathways, biofilm restructuring, and host–microbe metabolic signaling. Overall, current evidence supports a microbial ecological shift model, in which *Veillonella* participates in early dysbiotic transitions preceding OSCC development but may decline as tumor microenvironments evolve. Further standardized and multiomics studies are needed to clarify its potential as a microbiome-based biomarker and therapeutic target.

## 1 Introduction

**Figure 1:**
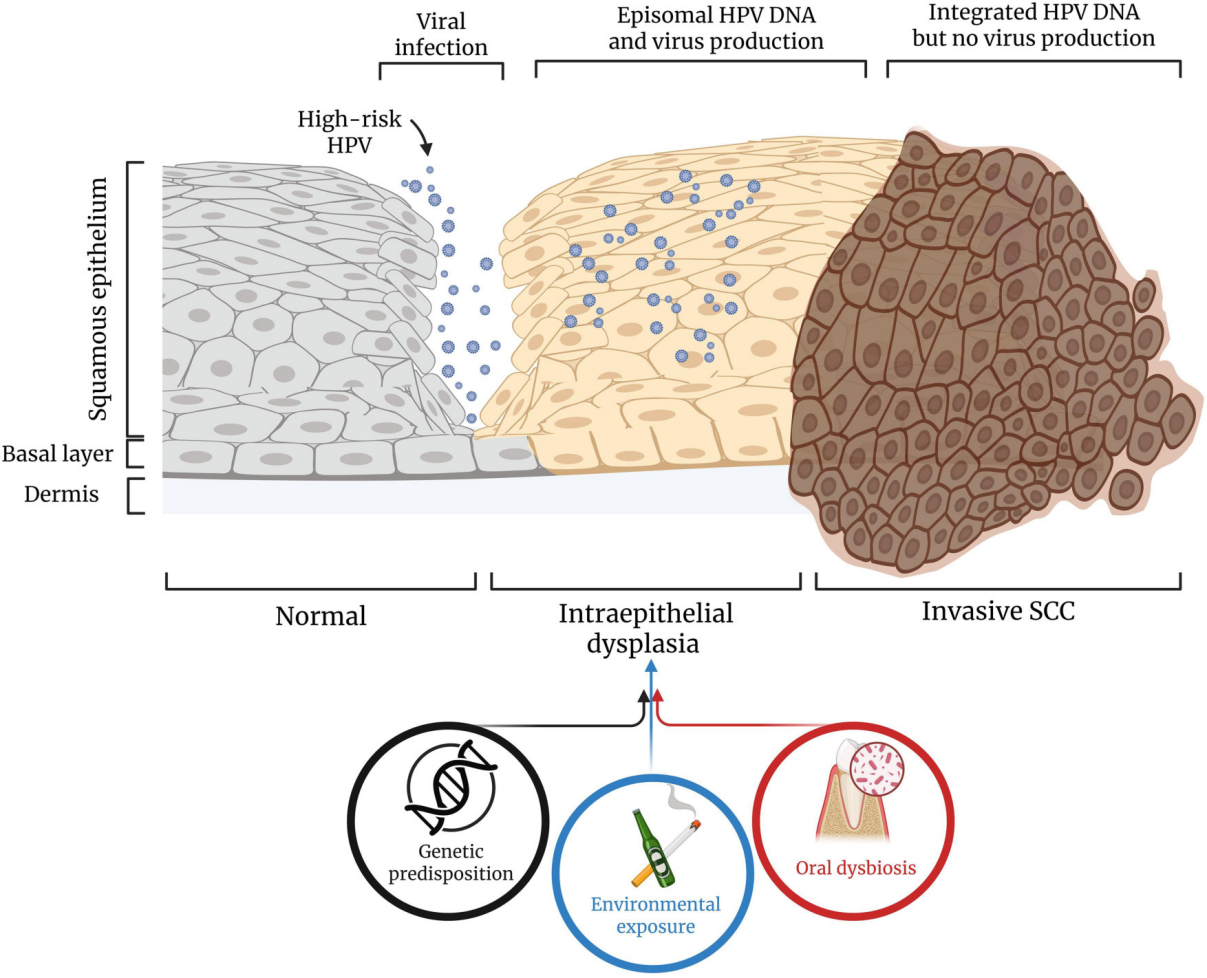
An overview of OSCC pathogenesis. A schematic overview of the multistep progression of OSCC and the contributing risk factors. The upper panel illustrates the sequential transformation of normal squamous epithelium into intraepithelial dysplasia and invasive squamous cell carcinoma. High-risk human papillomavirus (HPV) infects basal epithelial cells, where episomal HPV DNA supports viral replication and production. As dysplasia progresses, HPV DNA integrates into the host genome, halting viral production but promoting oncogenic transformation. The lower panel highlights the three major contributing factors that drive this progression: genetic predisposition, environmental exposure (e.g., tobacco and alcohol), and oral dysbiosis, which collectively induce molecular and cellular alterations leading to malignant transformation.

Oral squamous cell carcinoma (OSCC) is one of the most common malignancies of the head and neck region, posing a significant challenge to humans because of its aggressive behavior, complex etiology, and low survival rate (1). Most oral cancers are accounted for by OSCC, an invasive epithelial neoplasm. Although great strides have been made in medical diagnostics and therapies, the 5-year survival rate of OSCC still remains at approximately 50%, owing mainly to diagnosis in the advanced stage, recurrences, and a lack of knowledge about the molecular and microbial risk factors influencing the initiation and progression of this neoplasm (2). While risk factors such as tobacco use, alcohol use, and Human Papillomavirus (HPV) infection are well recognized, the oral microbiota has recently gained attention in OSCC pathogenesis (3). *Veillonella* species are one of the bacterial genera that have been a focus of recent studies to establish the association of the oral microbiota with oral carcinogenesis. The multistep progression of OSCC and its associated risk factors are summarized in Figure 1.

**Figure 2:**
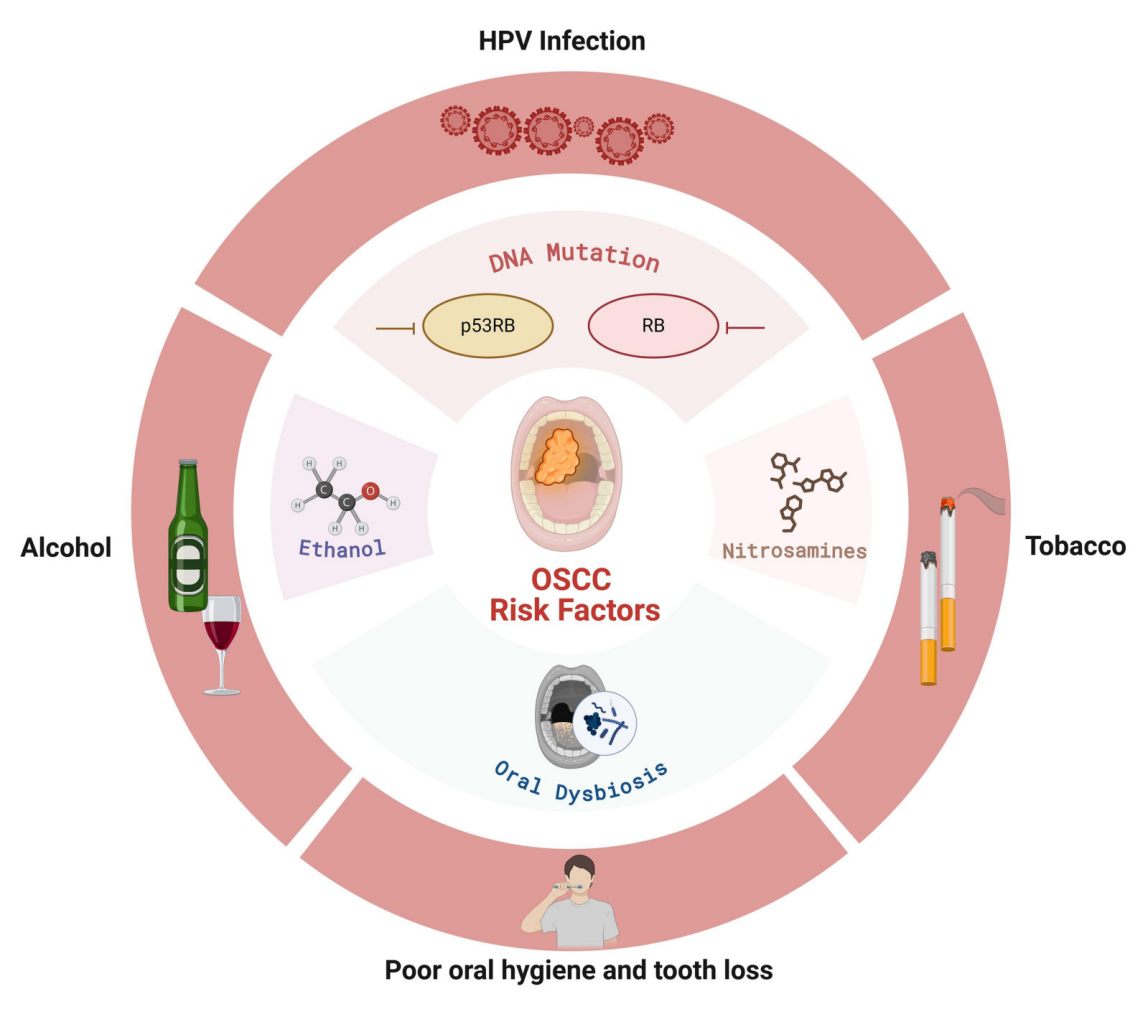
A schematic illustration of the major risk factors associated with OSCC and their relationship with oral microbiota. The diagram depicts how several etiological factors such as HPV infection, tobacco use, alcohol consumption, poor oral hygiene and tooth loss, and oral dysbiosis converge to facilitate carcinogenesis. Exposure to tobacco and alcohol results in the buildup of mutagenic compounds like nitrosamines and acetaldehyde, whereas HPV infection alters host tumor suppressor pathways (p53 and RB) through viral oncoproteins. Simultaneously, oral dysbiosis and chronic inflammation further alter the local microenvironment, promoting DNA mutations, epithelial damage, and microbial colonization that collectively increase OSCC risk.

According to the latest information of expanded Human Oral Microbiome Database V4 (https://www.homd.org/) (4), the oral microbiota consists of over 800 different species, which are present in a complex and diverse ecological community, playing several roles to promote oral and systemic health. On the other hand, the disruption of the oral microbiota, called “oral dysbiosis,” is implicated in several oral diseases ranging from periodontal disease and caries to OSCC (5). The interplay between major OSCC risk factors and oral microbiota, highlighting their combined contribution to carcinogenesis, is summarized in Figure 2. *Veillonella* species are strictly anaerobic Gram-negative cocci that are highly abundant in the oral cavity (6). Their main ecological functions are based on the metabolism of lactate, which helps in pH stabilization. Previously regarded as commensal and non-pathogenic members of the human microbiota, *Veillonella* spp. have now been associated with a vast range of oral health states (7).

Recent studies have revealed significantly different levels of *Veillonella* within the oral cavities of patients with OSCC in comparison with healthy controls (8), thus focusing on the possible association of this bacterial genus with oral carcinogenesis. Because of its role in chronic inflammation, immunity, and production of metabolic products, the impact of *Veillonella* on the oral microenvironment may contribute to crucial carcinogenic processes. *In vitro* and *in vivo* assays suggest that certain *Veillonella* metabolites and molecular interactions could promote tumor formation and progression by enhancing cell proliferation, angiogenesis, and immune evasion (9). There is emerging evidence that *Veillonella* also contributes to genetic and epigenetic alterations of oral epithelial cells (10). Therefore, this narrative review assesses the scientific evidence available in the literature concerning the prevalence and role of *Veillonella* spp. in OSCC. The main research question is: What effect does the prevalence of *Veillonella* species have on the molecular mechanisms and clinical outcomes related to the incidence of OSCC? The review will mainly focus on clinical, molecular, and epidemiological studies to both understand the prevalence of *Veillonella* and investigate the possible role of this genus in oral cancer. Also, the review will elaborate on the clinical relevance of *Veillonella* spp. in OSCC regarding its clinical usage as a biomarker and therapeutic target to control oral carcinogenesis.

This narrative review synthesizes available evidence from clinical, epidemiological, and molecular studies investigating the potential role of *Veillonella* species in OSCC. Relevant studies published in English were identified through searches of major scientific databases, including PubMed, Scopus, and Web of Science, using keywords related to *Veillonella*, oral microbiome, dysbiosis, and OSCC. The retrieved literature was critically evaluated and comparatively synthesized to identify dominant findings as well as similarities and discrepancies among existing studies. However, current research exploring the association between oral microbiota and OSCC remains limited and heterogeneous. Differences in microbiome assessment techniques, sample sizes, and methodological approaches across studies may contribute to inconsistencies in reported findings, highlighting the need for more standardized research frameworks in this field.

## 2 *Veillonella* species: overview and oral ecology

*Veillonella* spp. are key players of the oral microbiome, fulfilling important ecological and physiological functions within this ecosystem (11–13). Their unique structural and metabolic features make them important mediators of biofilm communities, and potential implications in diseases such as OSCC make them relevant when understanding the overall interplay between oral microbiota and oral carcinogenesis (14–18), which this work tries to address.

### 2.1 Taxonomy and general features

At the time of writing, genus *Veillonella* has 21 recognized species validly published under the ICNP (International Code of Nomenclature of Prokaryotes) (19). Among them, oral *Veillonella* has 11 species. *Veillonella* species emanate from the phylum *Bacillota* (20). They belong to the class *Negativicutes* and the *Veillonellaceae* family. Both *Bacillota* and *Negativicutes* are anaerobic and gram-positive or gram-negative, and therefore, they are somewhat phylogenetically related. However, the metabolism and cell structure of the *Veillonella* species diverge from that of *Bacillota* because the *Veillonella* species are strictly anaerobic and possess gram-negative cell walls. These distinct characteristics are important to understand the ecology of this genus in the oral cavity (6, 21–23).

As an anaerobic bacterium, *Veillonella* are round, or coccoid, in shape. These bacteria are not motile, and therefore, it is easy to distinguish them from bacteria of other genera in oral samples such as saliva, plaque samples, and tongue swabs (21, 23, 24). Unlike most other abundant bacteria of the oral microbiome, *Veillonella* species are not capable of fermenting any carbohydrates directly. Instead, they feed on lactate and other acids produced by initial colonizers like *Streptococcus* species (25). Therefore, *Veillonella* species are classified as early colonizers (26).

In most oral habitats, oxygen concentrations are not low enough to permit the exclusively anaerobic existence of *Veillonella*. However, under layers of biofilms, in dental crevices, or other low-oxygen habitats in the oral cavity, oxygen content diminishes sufficiently for *Veillonella* to thrive. With respect to dental biofilms, *Veillonella* colonize in the layers closer to the tooth and farther from the oral cavity because of their strict requirement for anaerobic conditions. In oxygen-poor conditions, *Veillonella* can participate in later stages of biofilm formation, which are important stages in the homeostasis of oral biofilm communities (25). Because *Veillonella* metabolize lactate, their effect on the dental biofilm is to reduce the local concentration of lactate, thus increasing pH in the microbial microenvironment. With less acid produced, enamel demineralization is decreased, reducing the risk of acid-related damage, like dental caries, in the area occupied by the biofilm. As an important buffer in oral biofilms, *Veillonella* species play an important role in controlling acid-related damage to the mouth (25).

*Veillonella* are found throughout the oral cavity, including dental plaque, saliva, and on the tongue (12, 24, 27, 28). They are abundant in both healthy individuals and those afflicted by oral diseases (29), highlighting their significance in general oral biology. Also, a study found that patients afflicted by OSCC demonstrated a significant decrease in the relative abundance of *Veillonella*. Furthermore, *Veillonella* showed decreased abundance in patients who underwent surgical treatment (30).

As an organism found in all oral samples, *Veillonella* may be important to maintaining a stable oral microbial community (11, 31). For example, in a recent OSCC study, the relative abundance of *Veillonella* was seen to be variable across different types of patients (i.e., cancer patients before any treatment, those after surgical treatment, etc.) compared with healthy control subjects (30). This indicates that the role and function of *Veillonella* may vary, which is not surprising considering their metabolic activity and their prevalence in oral biofilm formation (25). In addition, because of their ability to metabolize lactate, *Veillonella* can produce acetic acid and propionic acid in oral biofilms as metabolic end products (21). They prevent the lowering of pH within the biofilm, which allows other members of the biofilm community to flourish (23, 25).

*Veillonella* species are thought to be harmless commensals (23). However, they can affect disease processes within the oral cavity because their metabolic activity creates certain microbial microenvironments. For example, *Veillonella* produce catalase, which can alter gene regulation in *Fusobacterium nucleatum* (32). The consumption of lactate by *Veillonella* benefits oral biofilm stability by allowing anaerobic species to thrive (32). Apart from providing a growth-initiating factor, *Veillonella parvula* has been shown to produce heme, which is the preferred iron source, and which is also able to detoxify hydrogen peroxide produced by other early colonizing species through its catalase activity, facilitating the growth of less oxygen-tolerant anaerobes. The resulting stable, persistent biofilm may allow other disease-causing species like *Porphyromonas gingivalis* to survive (11, 26, 33).

It has also been hypothesized that *Veillonella* directly contributes to the tumor microenvironment. In patients afflicted with OSCC, it has been seen that the relative abundance of *Veillonella* is consistently low, even though they play an important role in maintaining microbial homeostasis in healthy individuals (30). This discrepancy may represent a shift in the ecological function of *Veillonella*, allowing diseases to progress more efficiently. In oral dysbiosis, which is the disturbance of microbial composition and ecosystem structure, the dysbiotic state facilitates disease (34).

Interestingly, studies have been inconsistent with regard to reporting variations in the relative abundance of *Veillonella* and OSCC. While many reports have illustrated a decreasing relative abundance for this bacterial genus as OSCC develops (30), some studies have also observed a slight increase in relative abundance with increased tumor aggressiveness (34). These inconsistent findings may be the result of varying standards of laboratory methods and analyses, which may produce vastly different results. Moreover, while several publications illustrate a decrease in the relative abundance of *Veillonella* in patients with OSCC, it was found that it mainly decreased only in those patients who had undergone surgery (30, 34–39). However, patients with OSCC had undergone different treatments prior to sample collection, including oral reconstruction, chemotherapy, and radiotherapy. These variations in treatment history may introduce inconsistencies that confound the interpretation of sample analysis. Such varying standards in relation to sample analysis confound the issue (40).

As such, the exact role of *Veillonella* in oral cancer is uncertain and may not be strictly categorized as protective, dangerous, or neutral. The varying standards with which studies have been conducted, the inability to differentiate between living vs. dead samples (i.e., living sample analysis), and the absence of direct mechanistic examinations render any conclusions about the effect of *Veillonella* on OSCC impossible. Furthermore, because *Veillonella* may promote the survival of more harmful species, the fact that its relative abundance is typically reduced in diseased oral tissues may only be an indicator of the shift toward disease. In this section, *Veillonella* was presented as a crucial and abundant species of oral biofilm communities. Yet, its role in the pathogenesis of diseases like OSCC remains obscure.

### 2.2 Role of *Veillonella* in oral health and disease

*Veillonella* species have been shown to have a major impact on the oral ecosystem, especially with regard to their cross-feeding interaction with other species in the oral microbial community. *Veillonella* uses lactate produced by cariogenic species like *Streptococcus*. By doing this, it indirectly stabilizes the pH levels of the oral environment, potentially buffering against enamel demineralization as it lowers the risk of cariogenic biofilms (41). *Veillonella* is a crucial secondary colonizer of oral biofilms. As *Streptococcus* species colonize, they will ferment carbohydrates, producing lactate. In turn, this promotes the growth of *Veillonella* and others. The consumption of lactate promotes a pH gradient away from extremes of acidity, thus making the oral environment more hospitable for non-aciduric species. *Veillonella* is an important part of this ecosystem. By lessening extreme acidification of the biofilm, *Veillonella* reduces the likelihood of caries. This helps prevent dysbiosis of the ecosystem and also provides resources for bacteria that cannot grow in highly acidic environments. Changes to *Veillonella* populations by the shifting conditions of microbial ecosystems may contribute to disease by creating dysbiotic situations.

The genus *Veillonella* can play a key role in promoting the development of healthy and diverse oral biofilms. Research studies support the ability of the genus to increase the stability of oral biofilms (11, 28). *Veillonella* influences the progression of dental caries because of its ability to utilize lactate. Some strains of *Streptococcus* produce lactate as a by-product from consuming carbohydrates, and if this lactate is not controlled by *Veillonella* consumption, caries may form (42). The ability of *Veillonella* to impact pH levels in order to regulate caries prevention may be context-dependent. It is impacted by environmental and microbial factors. Any changes in the complex interactions among the different species and strains of the oral microbial community could interrupt the positive impact of *Veillonella*, shifting from being helpful to harmful. Microbial imbalances due to dietary and host factors impact the ecology of biofilms (43).

The oral microbial composition will shift with the development of many oral diseases, including dental caries and periodontal disease. Clinical and molecular data show that *Veillonella* is elevated in caries-affected biofilms (41, 44). *Veillonella* has been shown to be an abundant member of the diseased oral cavity and participates in biofilm maturation (45). As an early colonizer, *Veillonella* contributes to the stabilization of both commensal and pathogenic microorganisms, promoting the growth of more mature and robust biofilms, ultimately causing chronic inflammation and the breakdown of host tissues, a common hallmark of periodontitis (6, 11, 12).

**Figure 3:**
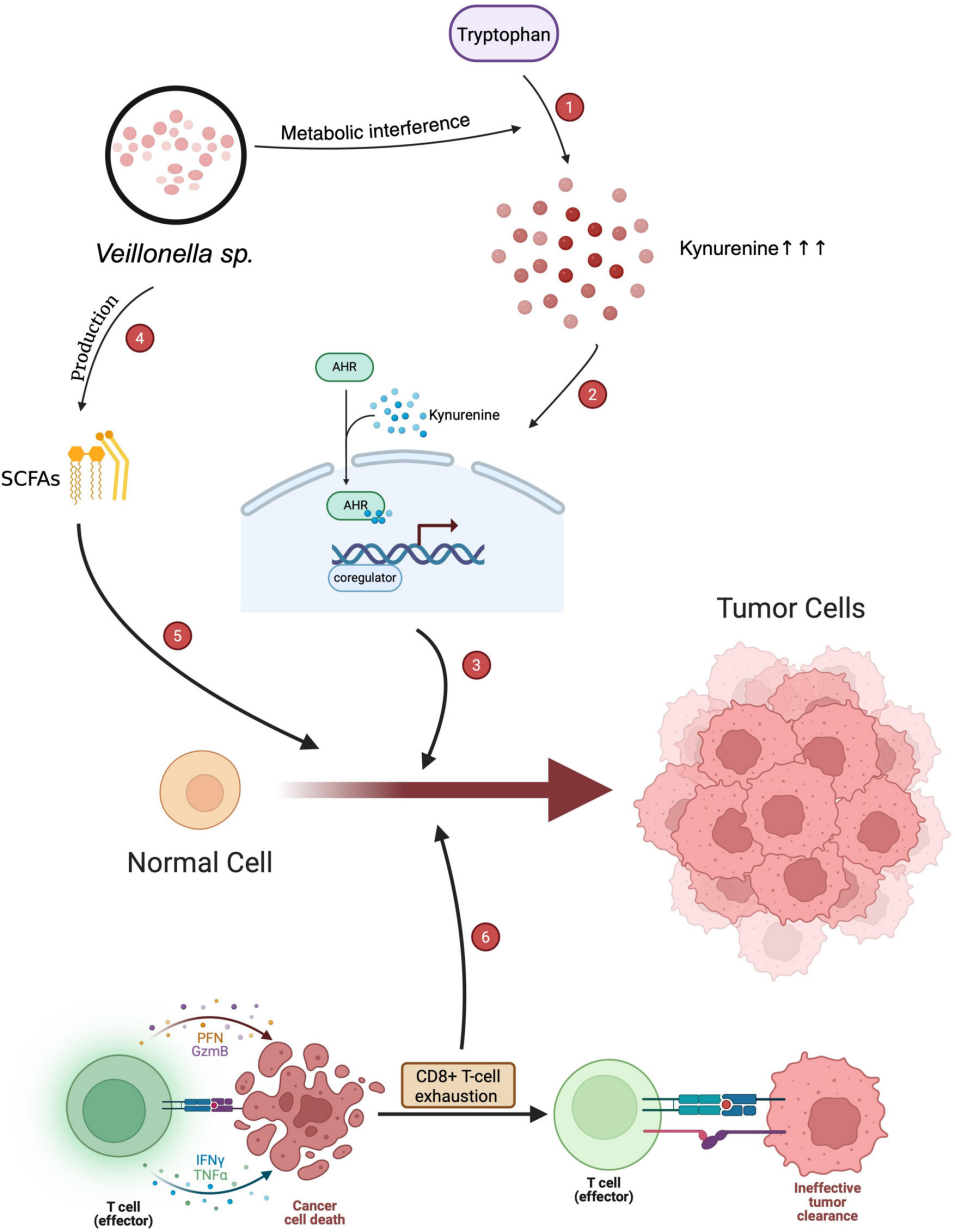
Hypothesized mechanisms of *Veillonella* species contributing to oral carcinogenesis. (1) *Veillonella* species disrupt the metabolism of tryptophan and result in increased kynurenine production. (2) Excess kynurenine stimulates AHR (aryl hydrocarbon receptor) signaling, resulting in transcriptional reprogramming toward tumor-promoting signaling. (3) This AHR activation drives tumorigenic transformation on the part of normal cells and contributes to tumor cell proliferation. (4) *Veillonella* species also produce short-chain fatty acids, or SCFAs, which can also modify host mechanisms of immune and metabolic responses. (5) SCFAs modulate immune homeostasis and promote the microenvironmental alterations promoting cancer progression. (6) Chronic inflammation followed by immune escape through CD8⁺ T-cell exhaustion is induced by tumor progression and leads to ineffective tumor clearance.

*Veillonella* can also produce metabolites toxic to the host. This may contribute to the pathogenicity of biofilms. One aspect of the negative impact that this genus can cause is the presence of volatile sulfur compounds and short-chain fatty acids (SCFAs). These by-products may damage the host tissues, interfere with epithelial barrier integrity, and have the potential to modulate other diseases (46–49). Emerging evidence suggests that *Veillonella* may extend its role beyond oral ecology to influence host-microbe interactions relevant to carcinogenesis, as illustrated in Figure 3.

Certain factors can cause an increase or decrease in the overall population size of *Veillonella* in the oral cavity. Oral environmental or host factors may contribute to shifts in the presence of this species within a dental biofilm. A high intake of sugars can increase the production of lactate by saccharolytic species such as *Streptococcus*, thus creating more nutrients available for the use and growth of *Veillonella* (50). A decrease in saliva flow can promote the growth of the genus, along with an increased intake of carbohydrates. A lower pH is another factor that may also contribute to the growth of the genus. Another factor, especially in periodontal biofilms, is the inflammatory environment of the oral cavity itself. This can promote shifts in microbial community composition because it impacts both oxygen and nutrient availability within the biofilm, shifting the composition of the microbial communities that thrive there (43).

**Figure 4:**
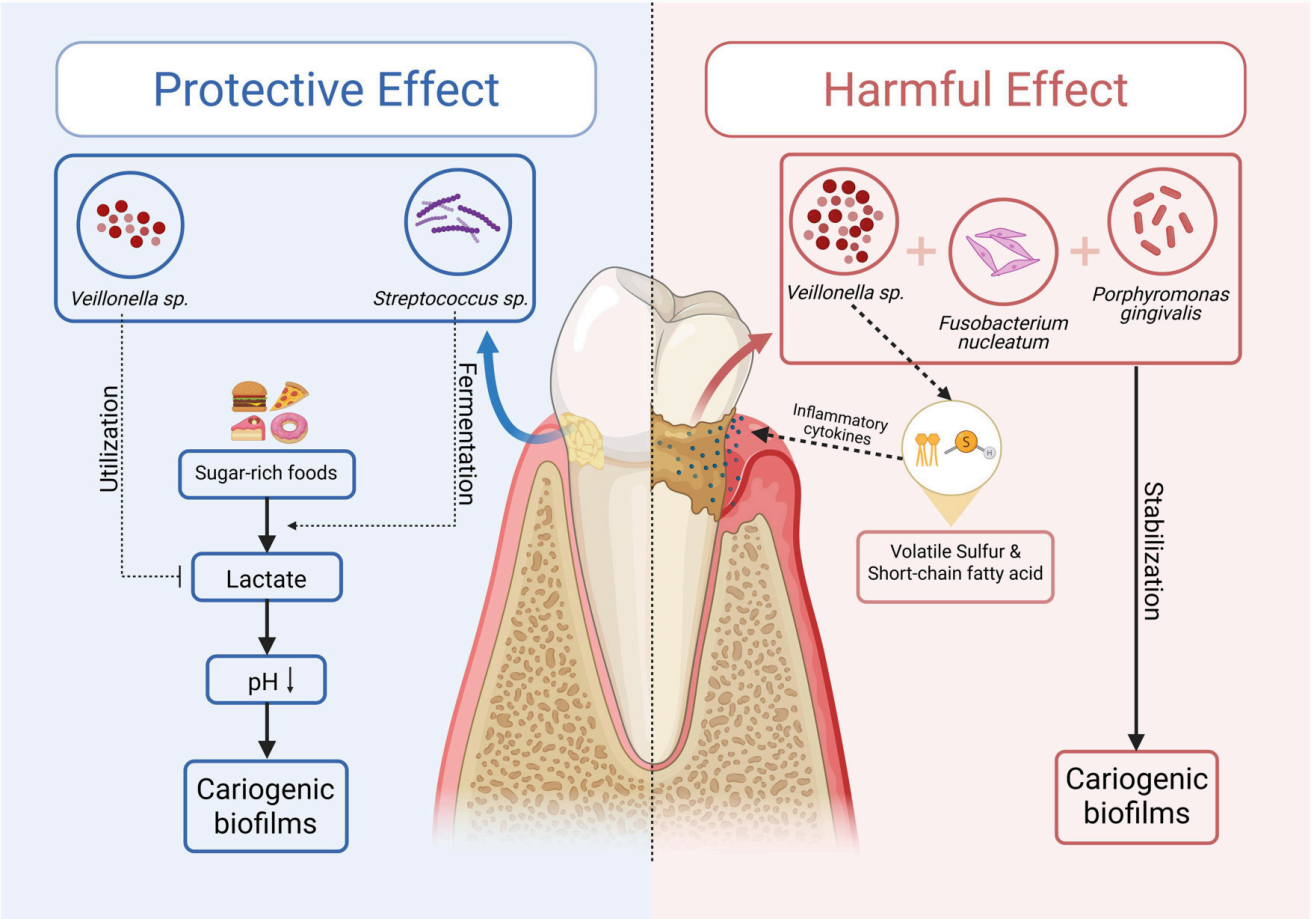
Ecological and metabolic roles of *Veillonella* in oral biofilms. *Veillonella* contributes to oral biofilm dynamics through its lactate metabolism, utilizing lactate produced by *Streptococcus* spp. to help maintain pH balance and support microbial stability under healthy conditions. In dysbiotic environments, however, its interactions with pathogens such as *Fusobacterium nucleatum* and *Porphyromonas gingivalis* may enhance biofilm maturation and promote the production of short-chain fatty acids and other metabolites associated with inflammation. These dual roles highlight the context-dependent function of *Veillonella* in maintaining or disrupting oral microbial homeostasis.

The role of *Veillonella* in biofilm maturation and cooperation with *F. nucleatum* and *P. gingivalis* create possibilities for it to be used as a therapeutic target (11, 26, 33, 51). Quorum sensing or custom-made antimicrobial peptides could be designed to disrupt those relationships to destabilize the biofilm structure and metabolic cross-feeding (11), which may reduce the growth of the protumorigenic environment created by biofilm dysbiosis. As with other interventions that involve bacterial targets, it is important to note that these interventions have to be carefully tested and have to target the specific microbial clusters responsible for driving biofilm dysbiosis, or they may risk inadvertently strengthening other interactions and microbial communities within the complex oral microbiome. These ecological and metabolic roles of *Veillonella* are shown in Figure 4.

## 3 Prevalence of *Veillonella* species in OSCC

Understanding the microbial landscape within the oral cavity is crucial for unraveling its role in OSCC development. The prevalence and distribution of *Veillonella* species across different clinical and biological contexts offer valuable insights into their potential as biomarkers and contributors to carcinogenesis. This section explores key clinical observations and factors influencing the presence of *Veillonella*, situating these microbial dynamics within the broader framework of oral health and disease progression.

### 3.1 Clinical observations and studies

Consistently, altered abundance patterns in the oral microbiome of individuals with OSCC have been highlighted for *Veillonella* species through clinical observations and studies, as shown in Table 1. A general reduction in *Veillonella* prevalence among cancer patients compared with healthy controls is indicated by research data gathered from saliva and tissue samples, as demonstrated by Zhang et al. (38) and Mougeot et al. (53). The potential disruption of microbial homeostasis in the oral ecosystem during OSCC development is underscored by this observation. It is suggested by this reduction that *Veillonella*, which is typically a stabilizing component of the oral microbiome, may lose its regulatory influence, thereby contributing to cancer-associated dysbiosis. To unravel whether the reduction is merely a consequence of carcinogenesis or a contributing factor to the disease, further investigation is needed, which is necessitated by the inverse association between *Veillonella* abundance and OSCC.

**Table 1:** Summary of studies investigating *Veillonella* prevalence in patients with cancers of the oral cavity.

Study	Year	Study model	Sample type	Methodology	Population/context	Main findings
Zhang et al. (38)	2020	Human (case–control)	Tumor tissues and saliva	16S rRNA sequencing	Adult OSCC patients vs. healthy controls; age not reported	Reduced *Veillonella* abundance correlates with advanced OSCC
Unlu et al. (34)	2024	Human (case–control; treatment-naïve)	Saliva	16S rRNA metagenomic sequencing	Adult OSCC patients (6M/4F) vs. controls (6M/6F); HPV-negative; Turkish cohort	Decrease of *Veillonella dispar*
Lou et al. (52)	2025	Animal model (mouse; GF and conventional, chronic stress model)	Saliva	16S rRNA metagenomic sequencing	Female 5-week-old mice under controlled experimental conditions	*Veillonella* associated with immune escape mechanisms in chronic resistant stress mice
Mougeot et al. (53)	2025	Human (clinical cohort; HPV-stratified)	Saliva	16S rRNA metagenome sequencing	Adult patients with HPV-positive vs. HPV-negative OSCC; age not reported	Increased *Veillonella* atypica in HPV-positive early-stage oropharyngeal cancer patients with OSCC

 Reports of decreased abundance of *Veillonella*, which is associated with OSCC, are not confined to a single study or cohort. Diminished *Veillonella* levels both before and after the surgical removal of tumor tissue were reported by Granato et al. (39), for example, indicating the persistence of microbial alterations beyond the physical presence of the tumor. This persistence suggests that the changes are influenced not only by the tumor mass, but potentially by systemic or localized disease-related factors such as chronic inflammation, immune response modulation, or metabolic shifts within the oral cavity (54).

The aforementioned findings raise a question whether the restoration of microbial homeostasis, including the levels of *Veillonella*, may play a role in postsurgical recovery or long-term disease management. The integration of microbiota-based approaches into current therapeutic strategies may potentially result from exploring the above hypothesis. Within the broader ecological shifts of the oral microbiome during OSCC, the observation of reduced abundance of *Veillonella* must be contextualized. It was in the context of a network of microbial changes, where the loss of commensal species coincides with the proliferation of opportunistic or pathogenic bacteria, that Mougeot et al. emphasized the depletion of *Veillonella* (53). Biofilm architecture, metabolic activity, and the microenvironment may all be influenced by such community alterations, ultimately fostering conditions conducive to malignancy. Taking into account the entire microbial ecosystem rather than focusing on *Veillonella* in isolation will help unravel the interconnectedness between *Veillonella* depletion and this ecosystem. It may also reflect broader shifts in the microbial community, where *Veillonella* is replaced by other taxa, making the ecological landscape in OSCC more complex; therefore, the observed reduction in *Veillonella* levels may not necessarily indicate its true depletion.

An intriguing connection between *Veillonella* levels and the clinical parameters of OSCC has also been observed (55). An inverse correlation between *Veillonella* abundance and indicators of OSCC severity, such as tumor size, lesion extent, and disease stage, was identified by Granato et al. (39). In this context, it is suggested that lower *Veillonella* levels may serve as a proxy for advanced disease progression, thereby introducing the possibility of using *Veillonella* as a prognostic marker. As tumors progress from early to advanced stages, the decrease in *Veillonella* prevalence reinforces the potential utility of microbial markers in evaluating tumor dynamics. Whether the decline of *Veillonella* levels actively influences disease progression or passively reflects underlying pathophysiological changes is unclear; therefore, further research is warranted to answer this question.

This phenomenon could be linked to residual inflammation, alterations in immune responses, or changes induced by therapy itself. That microbial restoration strategies might play a role in postoperative care, where monitoring and potentially rebalancing the microbiome could optimize recovery and reduce recurrence risk, is indicated by the findings of studies (56). In the postsurgical setting, the complexity of microbial succession and interactions necessitates targeted studies to further delineate these processes.

Because of divergent findings within the oral microbial community, the dynamics of *Veillonella* prevalence in OSCC are further complicated. A significant reduction in *Veillonella dispar* was observed in oral cancer patients, while other genera, such as *Gemella haemolysans*, exhibited increased levels, as noted by Unlu et al. (34). This coexistence of enrichment and depletion among different taxa suggests that there are competitive or compensatory interactions at work. For instance, the ecological niche vacated by *Veillonella* may be occupied by opportunistic bacteria like Gemella, which may reshape the biofilm to promote tumorigenic processes (57). The narrative of a singular causative relationship between *Veillonella* and OSCC is challenged by these microbial successions, and instead, a system-level perspective that considers the multitude of interactions within the fluctuating microbiome is advocated.

Species-level nuances within the *Veillonella* genus similarly complicate its relationship with OSCC further. *Veillonella atypica* was identified by Zhang et al. (14) as one of the differential markers in OSCC-associated dental plaque and saliva samples, indicating variability in how individual species within the genus may contribute to or reflect disease processes. Certain *Veillonella* species may exhibit unique ecological or functional roles depending on the oral niche and disease context, which is implied by this intragenus diversity. Examining microbial populations at the species level rather than generalizing genus-level trends, particularly when developing diagnostic or prognostic biomarkers, is also stressed by study findings

When considering the interplay between *Veillonella* and external factors such as viral coinfections, further complexity arises. Between changes in *Veillonella* abundance and viral infections, such as Herpes simplex virus 1, significant associations were reported by Zhang et al. (58), suggesting that microbial–viral interactions may jointly influence the progression of OSCC. Immune dysregulation may be amplified by these interactions, which could further disrupt the oral microbiome. This evidence highlights the multifaceted nature of OSCC-associated dysbiosis and the need to incorporate microbial and viral interplay into future research frameworks.

Across diverse oral diseases, including gingival squamous cell carcinoma, periodontitis, and healthy controls, microbial changes have been compared by broader studies, such as those by Li et al. (35). Not unique to malignancy, shifts in *Veillonella* composition may also occur in other chronic inflammatory conditions, as indicated by these comparisons. Generalized dysbiosis rather than disease-specific changes may be reflected by *Veillonella* alterations, which complicates efforts to define its role in OSCC, and such findings suggest this. Variability in *Veillonella* prevalence is further revealed by sampling from multiple oral sites, underlining the importance of site-specific analyses in understanding its ecological role.

**Figure 5:**
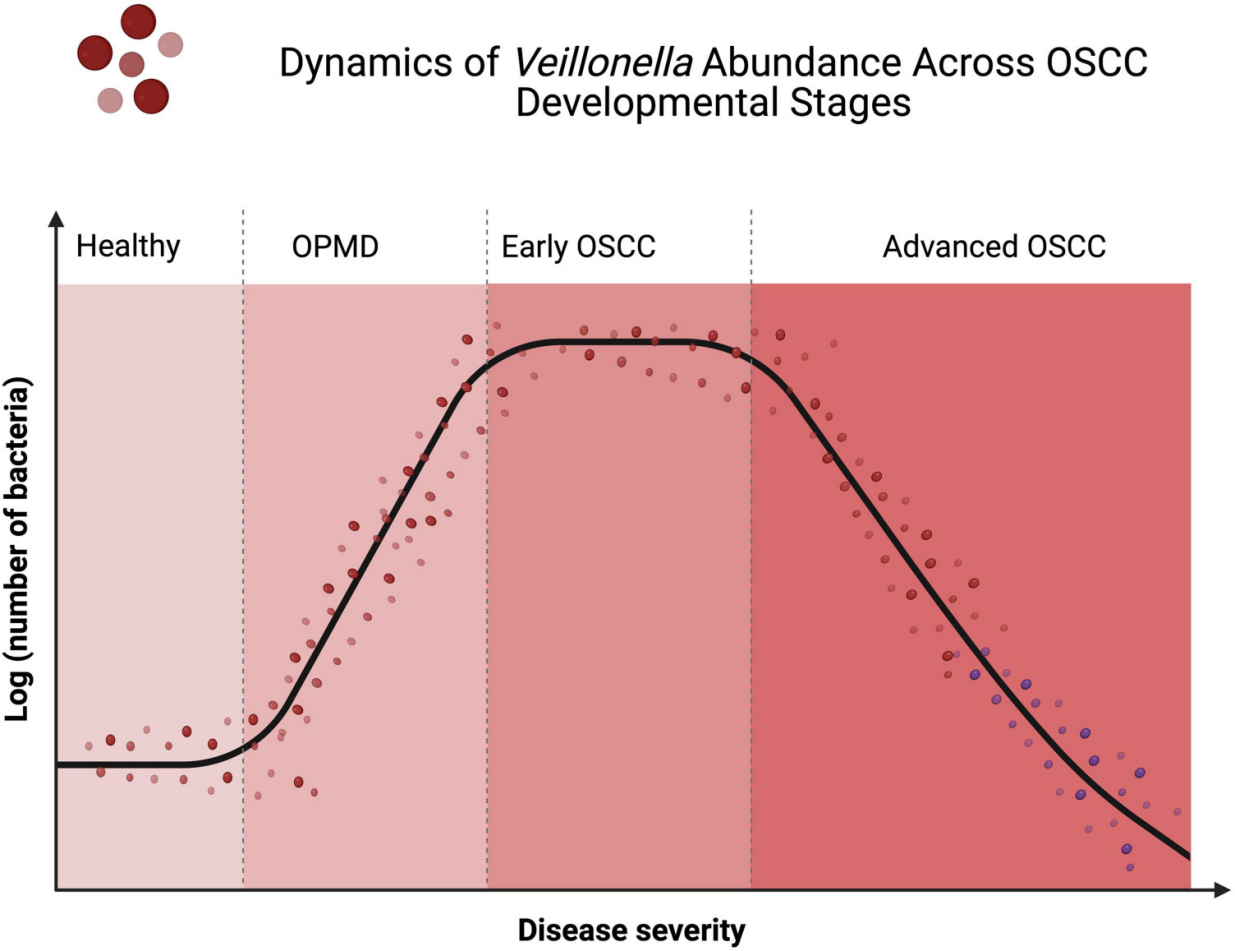
Dynamics of *Veillonella* abundance in OSCC developmental stages. The curve is a plot demonstrating *Veillonella* (log scale) abundance during progression of the disease from healthy mucosa to oral potentially malignant disorders (OPMDs), early OSCC, and advanced OSCC. The abundance of *Veillonella* increases in early dysplastic and malignant transformation stages but decreases in advanced OSCC, indicating that this early opportunistic colonizer does not proliferate over time as tumor-associated microenvironments become more oxygenated and less favorable for anaerobic growth.

Mechanistic insights into how changes in *Veillonella* abundance might influence oncogenic processes have been provided by Li et al. (37). That changes in the oral microbiome, including *Veillonella* alterations, may hold preventive or therapeutic potential is also suggested by existing literature. For interventions aimed at restoring balance and mitigating OSCC risk, the disruption of microbial homeostasis could serve as an early warning sign, as proposed by Sun et al. (59). However, a transition from observational studies to mechanistic and interventional research is required to leverage these insights. Whether *Veillonella* reduction prevents or accelerates malignancy remains a critical question, with broad implications for both diagnostic strategies and therapeutic development in OSCC. These dynamics of *Veillonella* abundance in OSCC development are illustrated in Figure 5.

The complex and multifaceted role of *Veillonella* in OSCC is highlighted by the collective body of evidence, ranging from potential biomarker utility to active involvement in oncogenic pathways. However, in disentangling its precise contribution from broader ecological changes and systemic influences, significant challenges remain. Ecological, molecular, and clinical perspectives must be integrated by future research to fully elucidate the relevance of *Veillonella* in the pathogenesis and management of OSCC, thereby providing a comprehensive framework for understanding its dual role as a stabilizer of health and a potential facilitator of disease.

### 3.2 Factors influencing *Veillonella* prevalence

The prevalence of *Veillonella* within the oral cavity is affected by a complex array of environmental and lifestyle factors that may disrupt microbial balance and increase susceptibility to OSCC, as shown in Table 2. Tobacco smoking represents one of the major factors affecting microbial composition of oral biofilms. Bataineh et al. (62) found that in comparison with non-smokers, smokers have significantly reduced the prevalence of *Veillonella*. Despite the clarity of these results, more studies are needed to understand whether lower *Veillonella* abundance is caused by direct toxicity of tobacco, changes in nutrient availability, and immune modulation and whether smoking cessation results in reestablishing *Veillonella* in smokers.

**Table 2:** Factors influencing *Veillonella* prevalence and their potential impacts.

Factor	Effect on *Veillonella*	Potential impact on OSCC	References
Tobacco smoking	Decreased abundance	Dysbiosis and chronic inflammation	(58)
Chronic restraint stress	Increased relative abundance	Immune dysregulation and microbial imbalance	(52)
Diet (high sugars)	Initially increased	Modulate inflammatory responses	(60)
Poor oral hygiene	Decreased beneficial commensals including *Veillonella*	Chronic inflammation and increased carcinogenic biofilms	(61)

Chronic psychological stress is another factor affecting *Veillonella* prevalence through its influence on host immunity and oral microbial balance. Several studies report that stress can lead to alterations in the immune system and promote colonization of opportunistic microbes, demonstrating that chronic stress enriched for *Veillonella* species may predispose for early neoplastic transformation. Interestingly, the studies show contradictory effects on several other taxa of *Veillonella*, suggesting that chronic stress–induced inflammation leads to the proliferation of specific *Veillonella* types under certain conditions. Additional longitudinal studies are required to test whether stress reduction is associated with an improvement in microbial balance within the oral environment (52, 63).

Diet also contributes to changes in *Veillonella* prevalence by altering microbial interactions. *Veillonella* is a bacterial genus that utilizes lactate produced by *Streptococcus* and other bacteria through fermentation of dietary sugars and other carbohydrates. The dietary preference for sugars may transiently increase *Veillonella*, since more lactate is available in the local oral environment (60).

Oral hygiene has also been implicated in *Veillonella* prevalence in patients with OSCC, since chronic periodontitis increases oral dysbiosis and oral cancer risk (33, 35). The interaction of *Veillonella* and periodontal pathogens like *P. gingivalis* may exacerbate dysbiosis, increase inflammation, and trigger cellular and genetic changes that can potentially promote tumorigenesis (33, 64). These results suggest that efforts to enhance oral health may improve *Veillonella* levels and reduce biofilm complexity in patients with oral cancer.

Often, the aforementioned factors occur simultaneously and synergistically influence *Veillonella* abundance. For example, smoking as a risk factor, together with poor oral hygiene, exacerbates the effect of each individual parameter, increasing the risk of oral carcinogenesis through dysbiosis (45). The combined effect of multiple risk factors could be incorporated in a multifactorial risk prediction model using quantitative measurements of *Veillonella* combined with assessment of behavioral risk factors such as tobacco use and oral hygiene practices. Multiplex oral microbiome analyses could provide a useful framework for personalized diagnosis and preventive strategies.

*Veillonella* levels change over time during the progression from oral health to OSCC and are influenced by factors such as dietary patterns, tobacco use, and oral hygiene, which shape its presence within the oral environment. For example, Granato et al. (39) reported that *Veillonella* abundance was significantly reduced in oral cancer tissues in comparison with controls without OSCC. This effect extends beyond tumor colonization, as *Veillonella* reduction in cancerous patients is observed prior to and following surgical excision of tumors. This outcome may be due to persistent systemic inflammation, immune suppression, or microbial destabilization resulting from tumor establishment or excision. Further studies are needed to test whether interventions with probiotics or microbiota-based therapeutics will restore microbial balance postoperatively.

The findings of studies are further supported by the positive correlation between clinical parameters such as tumor size and stage and *Veillonella* abundance, as reported by Granato et al. (39). The authors found that patients with smaller tumors and earlier stages of cancer displayed significantly higher *Veillonella* levels, and therefore, *Veillonella* levels are useful markers to define the severity of OSCC. However, whether the reduction of *Veillonella* prevalence influences tumor growth and clinical stage remains to be further elucidated.

Interestingly, studies have demonstrated the opposite effect of *Veillonella* prevalence along OSCC stage progression, and in different anatomical locations. Granato et al. (39) observed an increasing *Veillonella* prevalence from healthy tissue to epithelial dysplasia followed by decreasing levels when a tumor was formed. The presence of increased levels of *Veillonella* and other commensals might assist in creating a strong biofilm in early dysplasia, but as the oral cancer stage increases, *Veillonella* populations decrease because of the instability of the microbial community (34), allowing dysbiosis to occur. Further research using longitudinal experimental designs is warranted to test whether interventions aimed at manipulating *Veillonella* abundance are protective against or aggravate tumor formation at each stage.

Host-related factors that may contribute to the alteration of *Veillonella* prevalence in the OSCC oral environment include genetic predisposition, host immunity, and inflammation. Host genotype plays a key role in dictating the sensitivity of the host to microbial colonization (43). The inflammatory state found in OSCC tissues also influences the microbial composition of the oral cavity (35). High levels of inflammation significantly decrease oral health scores and reduce the level of healthy oral commensals, including *Veillonella*. Also, it is possible to allow more virulent and carcinogenic oral biofilms to thrive in the altered tumor microenvironment by decreasing pH, altering nutrient availability, and influencing host immunity. These changes ultimately influence host–microbe interactions, promoting local and systemic inflammation, as well as cancer promotion and growth (65). Further studies are needed to assess how personalized approaches targeting host genetics and the inflammatory profile might promote improved *Veillonella* persistence in patients with OSCC.

Methodological variations also play a major role when reporting *Veillonella* abundance across different studies. As microbial composition is highly diverse depending on the oral niche (66), the types of samples analyzed affect the levels of *Veillonella* in each study (saliva, oral mucosal swabs, tumor biopsies). Furthermore, several studies may use different DNA extraction techniques, 16S primers, sequencing platforms, analysis protocols, and taxonomic annotation.

There are also other limitations that can influence *Veillonella* abundance in the OSCC microenvironment. *Veillonella* prevalence may vary at different time points. *Veillonella* numbers may alter as an indirect result of chronic diet and changes caused by tobacco smoking. On the other hand, *Veillonella* abundance may differ from site to site in the oral environment, as well as during the progression from early precancerous lesions to fully established tumors (45). Interestingly, Zhou et al. (11) showed that the *Veillonella* genus was highly associated with another microbial genus, the lactic-acid-producing bacteria *Streptococcus*. *Veillonella* uses lactic acid produced by *Streptococcus* through carbohydrate fermentation to sustain cell growth and survival. According to this result, during cancer development, as dysbiosis occurs, lactic acid production decreases. Then, it is possible that *Veillonella* will grow in this niche. Overall, future experiments need to consider the temporal and spatial differences in microbial abundances and explore the ecological forces leading to these complex patterns.

In conclusion, the prevalence of *Veillonella* species is greatly influenced by environmental, lifestyle, host, and methodological factors. A deeper knowledge of these influences can lead to improved approaches to targeting oral microbiota to alleviate oral carcinogenesis.

## 4 Mechanisms linking *Veillonella* species to oral carcinogenesis

Overall, the literature reviewed in this study indicates that alterations in *Veillonella* abundance are consistently associated with OSCC, although the direction and magnitude of these changes vary across studies. Several reports suggest that *Veillonella* species may be enriched in early stages of oral carcinogenesis or in oral potentially malignant disorders, whereas reduced abundance has frequently been observed in advanced OSCC lesions. These contrasting patterns suggest that *Veillonella* may play a context-dependent role in the tumor microenvironment, potentially contributing to early dysbiotic shifts within oral biofilms through metabolic cross-feeding, immune modulation, and interactions with other microbial taxa. This section highlights the complex ecological role of *Veillonella* in oral microbial communities and supports the hypothesis that this genus may contribute to the dynamic microbial processes associated with OSCC development.

### 4.1 Chronic inflammation and immune response alteration

Chronic inflammation in the oral cavity is a pivotal process in the progression of OSCC, and microbial dysbiosis is thought to play an integral role in mediating these inflammatory processes. *Veillonella* species, a genus of bacteria commonly found in the oral microbiome, have demonstrated the ability to modulate inflammatory responses in the oral cavity, suggesting their potential to influence the development of OSCC (16). However, since the dual role of *Veillonella* species in inflammation can be both protective and harmful, depending on its presence in the oral cavity and the complexity of the microbial community, there is a need to further research these complex effects.

*Veillonella-*derived products such as lipopolysaccharides, short-chain fatty acids, and other metabolites can trigger pattern recognition receptors (e.g., TLRs) on immune and epithelial cells, leading to downstream cytokine release and modulation of immune cell recruitment and activation (67). These host–microbiome interactions may establish a permissive environment for cancer initiation and progression, and IL-6 and IL-8 are known to contribute to cancer pathways such ase angiogenesis and epithelial–mesenchymal transition, whereas TNF-α can lead to cell damage and microenvironment alterations, promoting cancer progression (68). Whether this contribution of *Veillonella* directly triggers OSCC or represents a symptom of a change in the bacterial community remains to be explored.

*Veillonella* species can affect epithelial and immune cell barriers. Lower abundances of *Veillonella* species in patients with OSCC suggest a deficiency in the defensive properties of these commensals, which may promote a permissive immune environment for cancer growth. This barrier-deficient property may not directly lead to the onset of OSCC but may simply make the body more susceptible to the disease and its progression. It is possible to consider that decreased levels of *Veillonella* species could lead to changes in barrier integrity, potentially enhancing exposure of the oral tissue to carcinogens, microorganisms, or microbial metabolites (10, 63). In addition, *Veillonella* species may serve to downregulate the apoptosis that provides antioxidative protection to cells, increase the number of T lymphocytes, induce cytokines (TNF-γ and TNF-α), and improve tumor suppression gene expression through lactate consumption in oral cavities (69). These can produce varying immune responses, some of which are immunosuppressive and thus promote cancer survival and dissemination. Further investigation may be crucial to evaluate *Veillonella* as an oral immune system influencer and an OSCC promoter.

*Veillonella* species can affect oral inflammation via the production of SCFAs (70). SCFAs are produced as metabolic by-products by several gut and oral bacterial species, including *Veillonella* species. Examples of SCFA molecules are propionate and acetate. SCFAs dampen local immune responses and create an environment of low-grade, prolonged inflammation, which is strongly correlated to cancer onset and progression (71). Furthermore, increased production of SCFAs may promote cancer onset by stimulating cell proliferation, while suppressing cell death or apoptosis in the cancerous cells, causing them to survive and multiply rapidly (48). Despite this, some studies have found that some SCFAs have anti-inflammatory properties, suggesting a complicated role for SCFA molecules in oral inflammation (70, 72–74). Therefore, the association between SCFAs and cancer remains largely unknown, requiring further research.

An animal model has been found to support *Veillonella* in playing a causative role in OSCC development in the presence of a dysbiotic environment, changes in metabolic activity, and immune evasion. Lou et al. (52) discovered that chronic restraint stress in murine models promoted dysbiosis in the oral microbiome, with *Veillonella* being upregulated. Increased kynurenine, a by-product from microbial metabolism enriched in dysbiosis, increased the activity of the aryl hydrocarbon receptor (AhR). This resulted in CD8+ T-cell exhaustion, which reduced antitumorigenic defense and promoted OSCC tumor initiation.

AhR, triggered by kynurenine, is a critical immunosuppressive and immune evasive pathway mediated by the increase of *Veillonella* populations in a dysbiosis environment. AhR activation by kynurenine during the onset of a tumor promotes T-cell exhaustion through increased secretion of inhibitory receptors by the adaptive immune system. Thus, the metabolism within the tumor microenvironment of *Veillonella* may promote systemic changes in the host to be more permissive toward tumor growth (34, 39, 52, 57, 65).

In general, the specific impact of *Veillonella* species on OSCC-associated inflammation remains to be uncovered. As of the writing of this document, the effects of *Veillonella* on oral epithelial and immune cells are mostly unknown. Currently, the existing publications merely reveal that *Veillonella* can modulate inflammation. The mechanism of *Veillonella* species in OSCC may be highly dependent on specific bacteria strains within this genus. Unfortunately, most articles have not explored specific strains within *Veillonella* that contribute most directly to OSCC in the oral cavity. Therefore, the overall role of *Veillonella* species in OSCC is not known. Further research must be conducted, as the currently available knowledge on the effect of *Veillonella* on OSCC cannot make claims to either protective or harmful functions on a person's overall oral health.

### 4.2 Metabolic and molecular interactions

*Veillonella* contributes to the metabolic microenvironment of the oral cavity by metabolizing lactate, a by-product of cariogenic and acidogenic bacteria, into SCFAs such as propionate and acetate (70). These SCFAs can act as signaling molecules, influencing cellular pathways within epithelial cells (10, 67, 75). In the context of OSCC, such pathways have been found to facilitate tumorigenic processes (10, 14, 67, 76). This implies that *Veillonella* metabolism may affect pH, redox, and other factors to influence the likelihood of DNA damage, dysregulated proliferation, or apoptosis of epithelial cells. The precise mechanisms through which *Veillonella* metabolism, in particular the production of SCFAs, can lead to OSCC still require further study.

SCFA metabolites of *Veillonella* influence signaling in the oral microenvironment. Propionate and acetate SCFAs have been implicated in affecting epithelial barrier integrity and anti-inflammatory and immune surveillance responses (77). Prolonged SCFA exposure has been found to trigger low-grade persistent inflammation in oral epithelial cells, increasing carcinogenic risk (46, 78). Low concentrations of SCFAs such as propionate may be necessary for anti-inflammatory signaling pathways and epithelial repair processes. However, higher concentrations of SCFAs in the dysbiotic oral microbiome appear to impair epithelial apoptosis and instead induce cellular proliferation, potentially facilitating the creation of tumors in oral tissues (77). These findings raise questions about the concentration-dependent effect of SCFAs derived from *Veillonella*, as well as the context in which SCFAs will prevent or induce tumor formation in OSCC.

*Veillonella* also has an impact on other oral microorganisms through its metabolic activity. Lactate is metabolized by *Veillonella* into SCFAs and changes competitive pressure for other bacteria (79). Through changes in competitive pressure and metabolic interactions with other bacteria, *Veillonella* creates possibilities to enhance the pathogenic potential of oral biofilms and facilitates the dominance of carcinogenic microbial communities (26, 41, 72, 80, 81). This metabolic impact can lead to the persistence of dysbiotic bacterial communities, creating stable communities that produce toxins and metabolites associated with tumor promotion.

Genomic and metabolomic studies indicate a correlation between the abundance of *Veillonella*, changes in the rest of the oral microbiota, and alterations in oncogenesis-linked gene expression in epithelial cells (14, 76). This relationship indicates a potential function for *Veillonella* as a biomarker of OSCC in early tumorigenic lesions such as leukoplakia. Specifically, such studies link *Veillonella* and changes in the remainder of the oral microbiome to changes in gene expression in terms of inflammation, immune cell function, the cell cycle, and programmed cell death. This strengthens the hypothesis that *Veillonella* metabolites may be a useful biomarker and even a driver of early carcinogenic changes. This hypothesis also suggests that *Veillonella* and the metabolic activity of its associated bacterial taxa are related to OSCC onset. However, more research into specific *Veillonella* metabolites and their impact on carcinogenic signaling in oral epithelial cells is needed.

*Veillonella* may be both protective and pathogenic for OSCC, depending on the ecological context. Dietary influences, other components of the microbiota, exposure to tobacco or alcohol, and other factors may have a crucial impact on how oral microbiome, including *Veillonella,* impacts OSCC (62). For instance, *Veillonella* may normally buffer against extreme acidic conditions and help maintain homeostasis of the oral microbiota (11, 31, 41, 60). This suggests that the interaction between *Veillonella*, the metabolism of its microbial consortium, and environmental factors is likely to impact whether *Veillonella* leads to protection or harm in OSCC, implying a context-dependent role.

The hypothesis of SCFAs directly affecting genetic expression is supported by the finding of the impact of tumor suppressor genes and methylation of the promoter sequence. Feitelson et al. (49) suggest that *Veillonella* alters tumor-suppressive mechanisms in OSCC pathogenesis. Woo et al. (82) found that increased SCFAs produced by bacteria such as *Veillonella* and chronic dysbiosis exposure have been correlated with decreased methylation in promoter sequences related to apoptosis, inflammatory response, and epithelial regulation. However, more high-resolution studies are needed to confirm the relationship between oral *Veillonella* abundance and specific host gene expression in OSCC.

By producing SCFAs as a waste product, *Veillonella* can disrupt the integrity of the epithelial barrier. When such breaches in the oral epithelium occur, carcinogens and other harmful microbes have the potential to breach tissue layers and cause epithelial damage, chronic inflammation, and eventually tumor formation (46, 78). *Veillonella* can change the balance of the immune environment locally as well. It is believed that long-term exposure to SCFAs such as acetate and propionate could exacerbate low-grade inflammation, increasing the likelihood of tumor formation (77). SCFAs influence the levels of inflammatory cytokines in tumor-infiltrating cells in OSCC (83). Chronic low-grade inflammation resulting from SCFAs has been found to amplify proinflammatory cytokines such as IL-6 and IL-8, which can activate downstream mechanisms associated with tumorigenesis. IL-6 enhances epithelial–mesenchymal transition, whereas IL-8 promotes angiogenesis, a process in which the tumor creates its own blood supply. Through mechanisms such as IL-6 and IL-8 upregulation, prolonged exposure to SCFAs has been linked to tumor formation in OSCC (49).

In a similar manner, matrix metalloproteinases (MMPs) facilitate cancer cell invasion and metastasis in the tumor microenvironment. Exposure to SCFAs causes decreased levels of cellular adhesion molecules and increased MMP expression, promoting cellular migration. *Veillonella* metabolism may promote cellular migration in OSCC by releasing SCFAs in the local microenvironment and promoting the activation of MMPs (49). *Veillonella* also has the potential to influence epithelial behavior through epigenetic changes. It has been found that SCFA metabolites such as propionate and acetate of *Veillonella* species can act as histone deacetylase inhibitors and modify the epigenetic control of genes related to epithelial cell maintenance and immune function (82). These epigenetic mechanisms can lead to further immune dysregulation in OSCC tissues. The epigenetic control of inflammation in OSCC has been described as a crucial mechanism associated with field cancerization.

*Veillonella* and other oral commensals can directly interact with the host, influencing the development and progression of OSCC. *Veillonella* can modify biofilms, influencing the development, maturity, and maintenance of oral biofilms. *Veillonella* is also thought to be crucial in stabilizing and maintaining mature oral biofilms because of its bridging capabilities between other microbial species that enter the biofilm at different times (early and late colonizers). *Veillonella* can help connect early *Streptococcus* colonizers in the formation of multispecies biofilms in conjunction with the more opportunistic and pathogenic species, such as *F. nucleatum*, within oral microbial communities (84). By contributing to bridging among diverse bacteria, *Veillonella* can help stabilize biofilms. These stabilized and mature oral biofilms are more resistant to chemical or antibiotic treatment. Moreover, by helping create a niche for *F. nucleatum* to survive and reproduce (85), *Veillonella* creates possibilities to indirectly damage the integrity of the oral epithelium by facilitating the production of toxins from other species. This mechanism enhances cancer risk, tumor development, and tumor progression by increasing inflammatory processes, enhancing local DNA damage, or accelerating the turnover of cellular proliferation. *Veillonella*-mediated metabolic cross-feeding may contribute to this process because *F. nucleatum* cannot metabolize lactate directly. Through coaggregation and cross-feeding of metabolites, *Veillonella* species and *F. nucleatum* can interact to potentially influence OSCC (43, 45, 85).

By comparing differences between *Veillonella* in the initial phases of carcinogenesis, its influence on OSCC tumor formation can be better studied. By examining the abundance of *Veillonella* and its function in patients with leukoplakia, researchers aim to show how the effects of *Veillonella* on microbial metabolism contribute to OSCC. Preliminary research on *Veillonella* and the effect on precancerous oral lesions has begun to investigate the relationship between the abundance of the genus and the progression to OSCC. Preliminary findings have implied a positive association between *Veillonella* abundance and oral leukoplakia incidence. These studies included Mendelian randomization methods, showing genetic causality between *Veillonella* abundance and development of leukoplakia in human patients (86), although more longitudinal studies and experimentation are required.

*Veillonella* has been implicated in OSCC progression because of its effects on host immune response, microbial metabolism, and its interactions with other microbial community members. Because of the ecological implications of *Veillonella* within the oral microbial consortium, a great deal of research is needed to uncover the exact role played by it in human health, with more work on the specific and causal mechanisms of *Veillonella* metabolism.

### 4.3 Potential genetic and epigenetic alterations

The potential genetic and epigenetic changes caused by *Veillonella* species in OSCC may occur through its ability to indirectly affect the regulation of host genes through the production of metabolites such as propionate and acetate. These SCFAs produced by *Veillonella* diffuse to host tissues and directly affect the transcriptional control of major signaling pathways involved in tumor immunity, proliferation, and antiapoptotic responses.

SCFAs of *Veillonella* may affect inflammation and proliferation in OSCC. Studies have proposed that SCFAs produced by the oral microbiome may regulate expression in genes and pathways that could induce cancer initiation, chronic inflammation, and immune evasion (48, 49, 71). These pathways affect inflammatory mediators and tissue-modifying proteins. Feitelson et al. (49) highlight the controversy of SCFAs in their ability to be pro- or anti-inflammatory depending on concentrations and contexts. Thus, more research is needed to elucidate the precise role of SCFAs in maintaining tissue homeostasis.

The chronic inflammatory and immunomodulatory response that develops following microbial exposure in the dysbiosis oral ecosystem by the species of *Veillonella* may have epigenetic effects on host epithelial cells. The role of dysbiosis-induced chronic inflammation in modifying host epigenetics has been linked to increased incidence of cancers (18, 82). Mivehchi et al. (63) stated that oral bacterial epigenetic modifications may affect the early development of OSCC by altering epithelial immunity and stability.

Various cross-sectional studies and clinical studies have highlighted a consistently inverse correlation between *Veillonella* species and markers for dysplasia in the oral cavity. Metsäniitty et al. (30) summarized an inverse correlation of *Veillonella* species with advanced clinical stage in OSCC. Hence, dysbiosis and *Veillonella* levels in the OSCC environment may provide a possible tool for future non-invasive early diagnostic and prognostic tests for OSCC.

Although there may be correlations of *Veillonella* population levels and genetic and epigenetic mechanisms in the OSCC ecosystem, causation still needs to be clearly established. The interplay of metabolic signals, inflammation mediators, and host genes may hold promise to target and prevent OSCC (45). High-resolution studies will be required to elucidate the specific host-oral microbiota signaling to help understand the causation and impact of the microbiome in OSCC.

The potential of *Veillonella* to manipulate the chromatin structure, host gene expression of DNA, and small non-coding RNA may provide a more biologically supported mechanism for its role in OSCC. However, future longitudinal studies are needed to address gaps that will help clarify whether the oral microbiome impacts the OSCC ecosystem. This will integrate molecular profiles in the oral ecosystem and the analysis of time in oral cancer progression to evaluate the interactions with *Veillonella* and the host to potentially identify targets to help prevent OSCC.

### 4.4 Mechanistic model linking *Veillonella* to OSCC progression

Although the precise role of *Veillonella* species in OSCC remains incompletely defined, accumulating evidence supports a context-dependent mechanistic model linking microbial metabolism, immune modulation, and tumor progression. Based on current clinical and molecular findings, we propose a hypothetical pathway through which *Veillonella* may contribute to OSCC development through metabolic and immunological interactions.

At the ecological level, *Veillonella* species function as key lactate-utilizing bacteria within oral biofilms (11, 42). By metabolizing lactate produced by primary colonizers such as *Streptococcus*, *Veillonella* generates SCFAs, including propionate and acetate (42, 79). These metabolites are not merely by-products but act as bioactive signaling molecules capable of modulating epithelial cell behavior, local pH, and redox balance (48, 75). In the context of dysbiosis, altered SCFA concentrations may disrupt epithelial homeostasis, promote low-grade chronic inflammation, and influence pathways related to proliferation and apoptosis (5, 65).

Beyond local metabolic effects, dysbiotic oral environments have been associated with increased activation of the kynurenine pathway, a key metabolic route involved in immune regulation (52, 87). Elevated kynurenine levels can activate the AhR, a ligand-dependent transcription factor expressed in immune and epithelial cells (87, 88). Activation of AhR has been shown to induce immunosuppressive signaling cascades, including the promotion of CD8⁺ T-cell exhaustion, thereby impairing antitumor immune surveillance (52, 88). This immunological shift facilitates immune escape mechanisms, allowing tumor cells to evade host defense systems and sustain progressive growth (65, 89).

Importantly, this mechanistic axis-linking *Veillonella*-mediated lactate metabolism, SCFA production, kynurenine accumulation, and AhR activationsuggests that *Veillonella* may act as an indirect modulator of the tumor microenvironment rather than as a classical oncogenic pathogen. In early stages of carcinogenesis, *Veillonella* may contribute to biofilm stability and metabolic buffering; however, under persistent dysbiosis, its metabolic outputs and interactions with other microbial and host pathways may shift toward a protumorigenic role.

This proposed model highlights a dynamic and stage-dependent function of *Veillonella* in OSCC, integrating microbial ecology with host immune and metabolic signaling. Future studies combining multiomics approaches such as metabolomics and immune profiling are essential to validate these pathways and clarify whether targeting this axis could offer novel diagnostic or therapeutic opportunities in OSCC.

## 5 Clinical and translational implications of *Veillonella* in OSCC

The role of *Veillonella* in OSCC extends beyond its ecological presence within the oral microbiome, pointing toward emerging clinical and translational relevance. Increasing evidence suggests that *Veillonella* may hold potential as both a biomarker and a modulator of the tumor microenvironment, offering new perspectives for early detection and risk stratification (43, 90, 91). At the same time, these insights open opportunities for microbiome-informed therapeutic approaches such as targeted modulation of microbial communities. Integrating microbiome-based understanding into broader cancer management strategies may ultimately support more personalized and context-specific interventions in OSCC.

### 5.1 Diagnostic and prognostic potential

*Veillonella* species are increasingly regarded as possible diagnostic and prognostic biomarkers in OSCC, and studies suggest that their abundance often varies in comparison with controls. For example, significant shifts have been observed in *Veillonella* species in both saliva and tissues of OSCC patients. Data generated via microbial profiling suggest that, for instance, *Veillonella* species are found in decreased abundance in OSCC (37, 45). However, methodological variations may contribute to incongruities across different studies, with differing sample processing and extraction protocols, in addition to the use of sequencing technologies, influencing the data obtained. Standardized diagnostic workflows are needed to ensure reproducibility across laboratories and to achieve effective clinical application.

For example, 16S rRNA gene sequencing data indicate that *Veillonella* species are often present in OSCC-affected saliva and tissue samples at lower levels compared with healthy controls (30, 45). As such, not only might reduced *Veillonella* abundance be indicative of OSCC, but the abundance levels might reflect aspects of the tumor microenvironment. Yet, results vary from study to study in their assessment of *Veillonella*, suggesting that some level of standardization is necessary to advance its assessment for clinical use. In this regard, standardized procedures may be required when acquiring samples to address possible variability in sampling techniques.

To validate *Veillonella* species as diagnostic biomarkers, multicenter and longitudinal studies that allow for cross-validation are paramount to their potential use in the identification of OSCC patients as well as to assess reproducibility across cohorts and at different stages of the disease. Using a broad range of patients and including large sample sizes are key to ensuring reproducibility of observations and for understanding whether potential *Veillonella* biomarkers are specific to different populations. Until these types of validations are put in place, *Veillonella* species abundance alone may not possess sufficient potential as a single, independent diagnostic marker. Moving forward with identifying which specific *Veillonella* taxa, if any, is of interest may also be crucial, given reports of enhanced *V. atypica* abundance in HPV-positive oropharyngeal cancer (53). The association between HPV and *Veillonella* may also suggest that *Veillonella* is colonizing an environment that is susceptible to such growth because of the changing oral microenvironment associated with viral infections (92). The potential use of *Veillonella* in the identification of distinct OSCC tumor subtypes may require its comeasurement with HPV status as well. While the exact mechanisms between HPV-induced immune changes and *Veillonella* colonization have not been elucidated, studies into the causation of microbial changes would assist with the identification of suitable biomarkers, perhaps through an examination of the immune environment and *Veillonella* in HPV-positive cells.

It is also interesting to note that changes in *Veillonella* abundance through the value of pH in oral acities are associated with differences in inflammatory cytokine expression in OSCC (45). Measuring the level of cytokines may, therefore, be a promising method of assessment to serve as a surrogate for immune response. These immune responses can also be indirect indicators of the tumor microenvironment and may serve as alternative clinical measures for tumor burden or stage. Moreover, by using inflammatory markers in conjunction with microbial markers, more informative and specific panels may be generated for OSCC diagnosis and prognosis. It is well documented that changes in the microbial landscape reflect various oral health states and conditions, including chronic periodontitis. For the utility of *Veillonella* species to be enhanced, incorporating these microbial profiles with inflammation markers of the epithelial response would allow for enhanced screening strategies for specific oral health ailments.

Given the fact that *Veillonella* prevalence decreases with increases in tumor size, tumor stage, and clinical grade (39), determining the potential of *Veillonella* in assessing disease progression may be a feasible strategy to use during and after treatment. Similarly, monitoring the progression from precancerous lesions to cancer is also valuable to patient treatment (37). This may be achieved by incorporating *Veillonella* species into other screening panels for oral health. Although *Veillonella* species prevalence may not be specific to OSCC, considering that similar decreases are seen in diseases like chronic periodontitis (11), there may be utility value when combining them with other microbial signatures such as *F. nucleatum*, which is often elevated in cancer tissues (53). Multiplexing microbial signatures with host markers to identify patients with OSCC may prove to be of utility value in patient assessment. For instance, the measurement of inflammatory cytokine profiles in saliva, together with the abundance levels of microbial species, may enhance OSCC specificity, and is more effective when they are used together as compared to using either assessment in isolation (45, 53). For such approaches to reach their true potential in clinical settings, further validation studies are required across broad-based, stratified cohorts in OSCC.

Technical difficulties may also hinder the utilization of *Veillonella* in the diagnosis or prognosis of OSCC. Differences in methods of DNA extraction from samples and differences in DNA primers and sequencing methodologies used across different research laboratories contribute to the variance in sequencing yields. As such, caution must be exercised when comparing results across multiple laboratories. These concerns make it hard to conduct a proper meta-analysis in the hope of identifying *Veillonella* diagnostic profiles with robust statistical power. To circumvent this problem, consensus guidelines may be necessary to ensure high levels of uniformity when applying sequencing platforms in different laboratories across the globe. Standardized methods are required at every step, from DNA extraction to bioinformatics pipelines. Inevitably, some variability will persist because of different methods that might be necessary to accommodate various technologies. Nonetheless, collaborative ventures and consistent communication may result in streamlined standards, leading to more reproducible results. This will be important, as the aforementioned issues could hinder translation into clinical practice.

The incorporation of *Veillonella* species as an adjunctive assay to molecular pathology techniques may assist in diagnosis and predict the responsiveness to treatment. Yet, studies evaluating *Veillonella* abundance in the diagnosis of OSCC must be evaluated and cross-validated. In addition, long-term monitoring is important to understand stability and sensitivity, which is crucial to ensuring effectiveness in a diagnostic environment. This will prove especially vital in the assessment of effectiveness of treatments on OSCC at specific locations. Combining the abundance of *Veillonella* species with epithelial levels of cytokines will add a host element to clinical assessment (45). The interplay between the inflammatory state of the epithelium and the colonization of specific microbial species provides the means for enhanced precision and may aid in personalized screening.

Nonetheless, while *Veillonella* might serve as a viable clinical option for assessment and diagnosis, this must be evaluated for clinical translatability. It can also serve as an excellent component of multiplexed screening tools used alongside other bacteria and host targets for oral cancers.

### 5.2 Therapeutic implications

The therapeutic potential of *Veillonella* species manipulation in OSCC treatment is of considerable interest, given their roles in oral biofilm dynamics and metabolic cross-feeding. The changes in *Veillonella* abundance observed in OSCC and oral potentially malignant disorders could offer therapeutic targets to manipulate the disease by modulating the overall oral microbiome structure. As Pignatelli et al. (45) hypothesized, by altering the biofilm architecture and reducing its metabolic cross-feeding capability, one could limit the growth of OSCC-promoting microenvironments. However, there is still much that needs to be uncovered with regard to exact therapeutic targets and mechanisms of action.

*Veillonella* can either be enriched in the tumor or reduced relative to healthy subjects or oral potentially malignant disorders, indicating that therapeutic windows might be limited depending on disease stage. Metsäniitty et al. (30) suggested that depending on the stage of disease, intervention strategies need to be different. Therefore, early-stage treatments could target imbalances and restore microbial communities to prevent OSCC transformation and tumor growth. Late-stage treatments would target the effects of dysbiosis. These interventions would have to be tailored to each disease stage, which needs to be understood in order to design efficacious therapies to disrupt dysbiosis within the oral microbiome of each individual.

Rather than completely eradicating all members of the *Veillonella* genera, it is worth pointing out that restoring the oral ecological balance is more realistic. As Pignatelli et al. (45) argued, microbiota intervention, combined with conventional OSCC treatment strategies, has the potential to be an effective and safe approach that may improve therapeutic outcomes, while sparing essential microbial benefits and minimizing the harm of dysbiosis. This approach avoids general broad-spectrum antimicrobial treatment that can promote antimicrobial resistance and unintended ecological imbalances, which may result in more aggressive microbial variants. Still, restoring the oral microbiome may require deeper insights into the intricate relationships of *Veillonella* within the ecosystem. In addition, understanding their influence in relation to host immune and epithelial cells and how these are altered in OSCC is important for therapeutic strategies that have long-term effects.

As for microbiota-targeted antimicrobial therapy and probiotic treatments for periodontopathic microbial biofilms in periodontal disease management, Santonocito et al. (93) pointed out that the decrease in certain disease-causing bacteria and reduction in host-inflammatory response were both able to reverse or prevent disease onset. This microbial shift toward a non-pathogenic state has been shown to decrease protumorigenic environments (90, 91). However, few studies in OSCC have investigated targeted antimicrobials and probiotics for *Veillonella* species modulation within the oral microbiome in OSCC. While targeting periodontopathogenic flora seems promising for periodontal disease, it is unknown whether these types of interventions will be similarly efficacious for targeting *Veillonella* and other bacterial species associated with OSCC. Interventions targeting microbial targets need to be designed at different levels of specificity (species, strains) and have to consider interactions with the host unique for each individual and their disease context.

Microbial interventions for individuals need to be personalized, considering individual microbial composition and inflammatory state in relation to the presence of disease. Santonocito et al. (93) mentioned that personalized approaches were more likely to result in desired microbiota modulation by reducing interpersonal variation. Personalized microbial therapies also can reduce unintended off-target effects because they are patient-specific. Large-scale clinical trials that incorporate sophisticated diagnostics that identify and quantify various bacterial strains, including *Veillonella* and their associated factors, will be crucial to determine the most effective approach for improving treatment outcomes.

Clinical intervention studies targeting the modulation of the *Veillonella* microbial cluster in OSCC have not yet been performed. For periodontal disease, there is promising evidence of combined adjunct interventions (probiotics, antimicrobials) on periodontal microbial community shifts after 3 months of conventional periodontal treatment of periodontitis in humans (93). However, OSCC is a distinct disease with differences in etiology and pathology compared with periodontal disease, and therefore, intervention studies need to be implemented and properly tested for feasibility and safety.

*In vivo* studies involving animal models have demonstrated that *V. parvula* colonizes the tumor for several days after its inoculation (94). Therefore, tumor colonization is a plausible therapeutic application of *V. parvula* as a tumor-targeting carrier in antitumor drug delivery. Yet, *V. parvula* does not seem to elicit any positive or negative tumor-promoting effects. Thus, in terms of a therapeutic benefit of suppressing *V. parvula* as part of the oral biofilm flora of OSCC in animal models, there is no clear conclusion. This suggests that genetic engineering of *Veillonella*, in combination with targeted delivery to the tumor microenvironment to release anticancer drugs or other therapeutic agents, could be a more viable therapeutic strategy than using them as is.

However, *V. parvula* does colonize in tumors for 7 days, which raises the concern of long-term ecological effects, and it poses safety concerns in immunocompromised patients. Long-term colonization in the tumor microenvironment may cause more damage than intended because of a broader disruption of microbial and immune cell populations (94). Preclinical studies that assess the safety profile of these live bacterial therapies *in vivo* for longer durations in OSCC are warranted. Given that a potential inverse correlation of clinical stage and *Veillonella* abundance was found by one group of researchers (39), *Veillonella* modulation interventions may only target *Veillonella* to exacerbate dysbiosis or suppress functions that are protective in some contexts.

Interventions aimed at microbiota modulation in OSCC can also be used as combined therapies. For example, modulation of microbiota and combined therapies such as surgery, radiation, and immunotherapy are all potential methods used to improve OSCC patient treatment. Collaboration between oral microbiome researchers and oncologists will be important for initiating such combined therapies. It is imperative to limit the negative off-target effects of such interventions on the oral microbial community. Pignatelli et al. (45) and Wang et al. (43) stressed that clinical trials are needed to test the efficacy of combining standard OSCC treatment with microbiota-targeting strategies. Clinical trials that investigate both strategies, in conjunction with clinical assessment data and advanced analytical methods, will provide critical information about the ecological features of dysbiosis associated with OSCC.

There is also a need for species differentiation to classify *Veillonella* based on their function. Diagnostic applications at species level will provide benefit in two ways: (1) species with pathogenic or protective functions can be targeted for intervention, and (2) species-level resolution can reveal more specific microbial dynamics involved in tumor progression (45).

Methodological constraints may be major barriers to the translation of *Veillonella* intervention techniques into clinical use. A major drawback is the absence of methodological standardization for microbiota analysis, due to inconsistencies among different OSCC and *Veillonella* research groups. A lack of standardized sampling, detection, and species definition results in poor resolution and difficulty in cross-comparing results from various laboratories.

Longitudinal, multicenter interventions involving different ethnic cohorts that examine both the health and the oral health status of patients, combined with a detailed analysis of the OSCC microbiome and tumor-site microbiome, are vital to developing safe and efficacious microbiota-modifying strategies. Furthermore, these longitudinal interventions must also adopt standardized methodological pipelines (sampling, data storage, bioinformatics), as mentioned earlier in this section. Standardization will also address the issue of methodological disparities in microbiome research. Wang et al. (43) called for studies of both microbiota-targeted prevention and treatment and studies of current treatment effectiveness incorporating a detailed microbiome analysis.

Species and strain characterization remains to be determined to pinpoint specific pathogenic and protective functions that *Veillonella* possesses. Insights into these specific functions can enable the development of rational intervention therapies that effectively target certain strains for either promotion or inhibition depending on what role they play in tumor progression, as suggested by Pignatelli et al. (45).

### 5.3 Proposed consensus framework for *Veillonella*–OSCC microbiome studies

Given the substantial heterogeneity observed across studies investigating *Veillonella* in OSCC, there is a critical need to establish a standardized methodological framework to enhance reproducibility, comparability, and translational relevance. Current inconsistencies in reported findings are largely attributable to variations in sample types (e.g., saliva, mucosal swabs, and tumor tissues), differences in DNA extraction protocols, sequencing platforms, and bioinformatics pipelines, all of which may significantly influence microbial composition and relative abundance estimates. To help address these issues, we propose a practical methodological framework that may support greater consistency in future microbiome research related to *Veillonella* and OSCC.

First, standardized sample collection should be prioritized, with clear differentiation between tissue-based and saliva-based analyses, as these represent distinct ecological niches and may yield divergent microbial profiles (8, 14, 95). Whenever feasible, paired sampling (e.g., tumor tissue and adjacent normal tissue, or saliva and tissue) should be implemented to provide a more comprehensive representation of microbial dynamics (14, 76, 95). Second, uniformity in DNA extraction methods is essential to minimize technical bias, particularly in relation to Gram-negative anaerobes such as *Veillonella*, whose detection sensitivity may vary depending on lysis efficiency (96, 97).

Third, sequencing strategies should be harmonized, including the selection of targeted regions (e.g., 16S rRNA V3–V4) or the adoption of shotgun metagenomics where possible, alongside adequate sequencing depth, to ensure reliable detection of low-abundance taxa (90, 98). Fourth, standardized bioinformatics workflows such as quality filtering, taxonomic classification and normalization approaches should be adopted to improve cross-study comparability. The use of validated and widely accepted pipelines (e.g., QIIME2, DADA2) is strongly recommended (96, 97, 99).

In addition, future studies should incorporate metadata standardization, including detailed reporting of clinical variables such as tumor stage, treatment status, oral hygiene, smoking habits, and dietary patterns, as these factors are known to influence *Veillonella* abundance and oral microbial ecology. Longitudinal study designs are also encouraged to capture temporal shifts in *Veillonella* populations during disease progression and treatment response.

By moving toward a more standardized and integrative research approach, future studies will be better positioned to clarify the ecological and functional roles of *Veillonella* in OSCC, ultimately strengthening its potential as a clinically relevant biomarker and therapeutic target.

## 6 Limitations and challenges in current research

In the area of *Veillonella* prevalence and OSCC, many methodological flaws must be addressed. Different samples were used across different studies. These samples include, but are not limited to, saliva, tissue biopsies, and subgingival plaque. Each sample represents a different microenvironment and thus a different microbial community. The varying abundance and community of *Veillonella* in different samples, for example, *Veillonella* in tissue biopsies vs. *Veillonella* in the saliva, make the comparison between these two sample types difficult (95, 100). Thus, there is no established standardized sample collection for the oral microbiome (30).

In addition, varied DNA extraction protocols and sequencing platforms are used across studies, both of which result in inconsistent and less effective results. Different primers used in 16S rRNA sequencing and sequencing depths further confound the identification and quantification of *Veillonella* species (12, 101). Studies have produced contradictory results. Some claim that *Veillonella* prevalence is elevated in precancerous lesions and decreased in OSCC (45), while other studies contradict these findings, stating that *Veillonella* is increased in OSCC (43). Therefore, a standardized set of procedures that may improve the reproducibility of results must be agreed upon.

The lack of standardized bioinformatics protocols in the literature also produces varied results. Inconsistencies in OTU clustering thresholds, analysis pipelines, and databases contribute to contradictory results. This results in varied taxonomic classification, leading to variations in the interpretations of the study outcomes (96, 102). Therefore, to ensure that results are consistent, reproducible bioinformatics pipelines, which have proven to be robust across studies, must be adopted by researchers. Without standardization, it is difficult to identify how changes in *Veillonella* prevalence associate with the development of OSCC and determine whether changes in *Veillonella* in the OSCC microbiome correlate to technical artifacts.

In most instances, case–control and cross-sectional approaches are applied in analyzing the oral microbiome. These techniques allow the prevalence and abundance of *Veillonella* to be assessed at only a singular point in time, impeding the establishment of the causal relationship between *Veillonella* and OSCC development. Thus, the causal relationship between *Veillonella* and OSCC must be more deeply explored to understand how *Veillonella* affects oral cancer progression.

Many publications reporting microbial research do not address diagnostic criteria (8, 55). No standardized thresholds exist for diagnosing abnormal levels of *Veillonella*, despite the extensive research surrounding its role in OSCC. This may be due to variable factors influencing *Veillonella* composition in the oral microbiome. These factors may include sequencing depth, selection of the bioinformatics pipeline, as well as differing levels of oral hygiene or differing diets across subjects. To enable the use of *Veillonella* species as a reliable biomarker in the clinic, standard parameters defining normal and abnormal levels of *Veillonella* abundance must be generated.

Furthermore, individual variations in demographics play a role in the variability of *Veillonella* in the oral microbiome and must be addressed. Variables such as age, sex, ethnicity, and socioeconomic status have demonstrated individual effects on the oral microbiome (97, 103), influencing the abundance, function, and community structure of microbes, including *Veillonella* species. Another gap in the research is the different units reported across studies. Differences in the reporting units used in studies can lead to varying interpretations. The literature is inconsistent, as some report results in terms of the relative abundance, while others use absolute values. When data are normalized, this has a marked effect on the values achieved. To make cross-comparison meaningful and easier, standard units for reporting bacterial abundances, which may be achieved with statistical normalization, must be utilized.

As the field progresses and more studies aim to quantify *Veillonella* prevalence to understand its role in the pathology of OSCC, the need for more selective and quantitative assays must be met. There is no single gold standard diagnostic approach that captures this bacterial prevalence. One method to bridge the gap in the detection of species is the design of improved sequencing platforms and optimized bioinformatics pipelines. Some current studies tend to contain relatively small sample sizes, thereby preventing these studies from achieving adequate statistical power for assessing associations between *Veillonella* and OSCC (45). More comprehensive, multicenter studies may provide more statistical power for *Veillonella* as a biomarker for OSCC in different populations. In addition, population size, tobacco use, alcohol consumption, and inadequate levels of oral hygiene (45) could serve as potentially confounding variables, because each factor independently impacts the bacterial community and increases cancer risk. Inadequate reporting of these confounding variables further complicates the comparison between these studies.

In addition, in several current studies, cohort samples were obtained and assessed in a single center or region. These cohorts could be subject to potential cultural or geographic influences that would affect the diversity of *Veillonella*. It must also be noted that there may be variations in *Veillonella* abundance in different environments and geographical locations because of regional dietary changes (13, 104). Moreover, with the use of cross-sectional and case–control studies, the literature lacks a robust mechanistic validation. Thus, the studies do not sufficiently demonstrate a causal effect of *Veillonella* on OSCC progression or address how *Veillonella* may trigger OSCC formation. Increased abundance of *Veillonella* in the cancerous or precancerous oral environment could be an effect, not a cause, of OSCC progression. Also, there is a lack of sufficient evidence supporting a causative role of *Veillonella* species in OSCC pathology and progression. Thus, prospective and longitudinal studies must be undertaken. The lack of research addressing *Veillonella* in the context of the oral cancer tumor environment may hinder advancement in this field.

A lack of translational frameworks in oral microbial research can also significantly delay translation into practice because studies do not integrate the fields of biology, chemistry, and microbiology to translate the research across the spectrum into clinical applications. It may also be challenging to extrapolate what effects of changes in *Veillonella* have been noted in oral microbes into the larger context of the oral tumor environment. Not linking biological effects and chemistry with microbial influences may delay the translation of research into clinical practice and limit clinical application. In addition, the biological characteristics of some microorganisms are difficult to translate into clinical applications. The integration and understanding of *Veillonella* biology and its functional properties are essential for translating this knowledge from bench to bedside and for the continued advancement of the field. Thus, future publications in this field should strive to integrate microbiology with biology and chemistry to achieve effective clinical translation.

Some taxa and strains of *Veillonella* can have conflicting effects on the inflammatory responses that may trigger carcinogenesis, while other types may have conflicting effects on bacterial growth, leading to the promotion or inhibition of biofilm growth. The abundance of certain taxa of *Veillonella* species has been reported to promote biofilm growth as well as carcinogenesis (11, 55). Future research is needed to address varying species-specific interactions between *Veillonella* species and their influences in cancerous and precancerous oral microenvironments.

Moreover, the exact impact of certain species of *Veillonella* is unknown. *Veillonella* species may have varied effects on the host and progression of cancer. More rigorous studies of different strains and different populations may elucidate species specificity and their exact role in disease pathology (105). In addition, the direct influences of the different species of *Veillonella* on different oral cancers must be investigated. There is also an absence of *in vitro* or *in vivo* studies to test or validate whether the presence or abundance of *Veillonella* is indeed responsible for carcinogenesis or for OSCC promotion, growth, or pathology. Future studies are recommended to test the impacts of *Veillonella* through manipulation such as depletion, enrichment, and transfer.

The gaps noted in microbial research resemble issues in other scientific fields. For instance, challenges that plagued the field of earthquake seismology involved methodological differences. These problems led to significant divergences in the results of seismic stress studies. A major issue concerned the application of inversion schemes and genetic algorithms in earthquake seismology, and the resolution was achieved by the application of both algorithms for the stress distribution problem. This demonstrated consistency in methodology, improved inversion, and robust solutions. Furthermore, the systematic application of data quality controls, together with both inversion schemes, yielded comparable and robust results.

The gaps identified above also lead to gaps in clinical understanding. For instance, with all the different methodologies in bacterial research, including techniques such as various sequencing and statistical methods, challenges persist in translating microbial research into clinical recommendations for diagnosis and/or treatment. If it indeed becomes difficult to translate this research into clinical use, then patients will not stand to benefit. Technical advancement of the field is limited because a thorough mechanistic and translational pathway, which advances scientific findings into patient application, does not exist.

To fully answer these questions and fill these gaps, investments must be made in new, high-resolution technologies, and in the long term, carefully coordinated epidemiological studies using similar populations and standardized technologies must be undertaken. The use of such longitudinal studies with standardized protocols and multiomics analyses may help identify specific molecular mechanisms through which *Veillonella* exerts its influence on the development or progression of OSCC.

## 7 Conclusion

The central purpose of this paper was to evaluate whether *Veillonella* prevalence in the oral cavity is correlated with OSCC progression and to assess whether it could be used as a biomarker, or a modulator, within the oral tumor microenvironment. This goal was accomplished by reviewing available clinical, epidemiological, and molecular investigations assessing *Veillonella* prevalence in health and disease, its ecological roles and interactions, its involvement in inflammatory and metabolic processes in OSCC, and its potential for use in the diagnosis and therapeutic treatment of this malignancy.

The main findings of this paper suggest that although *Veillonella* is a benign commensal bacterium, its abundance and behavior can be influenced by environmental cues, enabling it to act as both a protective and a harmful member of the oral microbiome. Clinical investigations frequently report a decrease in the abundance of *Veillonella* in advanced stages of OSCC, whereas they often show a shift in abundance toward an increase in this species in the potentially malignant oral cavity. These shifts have been linked to inflammatory and immunological conditions in the oral tumor microenvironment, microbial dysbiosis, and metabolic changes caused by *Veillonella* metabolites in OSCC. Available data suggest a potential use for *Veillonella* species as clinical biomarkers due to alterations in their abundance that are dependent on the clinical stage of the disease. However, current limitations prevent their broad implementation in cancer diagnostic and therapeutic procedures. Finally, it has also been shown that *Veillonella* species could play a role in OSCC by indirectly affecting the inflammatory milieu and directly impacting the tumors in the surrounding environment in association with other tumor-related species of the microbiome.

With regard to the broader context, it is clear that this analysis strengthens the notion that the oral microbiome plays a significant role in the etiology of OSCC. *Veillonella* species are often found altered along with other commonly implicated species such as *Fusobacterium* and *Porphyromonas* species, but the findings of this study indicate that these changes are species- and/or strain-specific and that *Veillonella* exhibits unique ecological roles, rather than indicating a passive shift in prevalence due to disease and oral dysbiosis. *Veillonella*, alone or in association with other species, could have the potential to actively impact the OSCC microenvironment and potentially impact host immunity against cancer, as well as act as a mediator of risk factor effects on the development of OSCC.

Although these are important conclusions and the findings support the role of *Veillonella* in the pathogenesis of OSCC, it is also important to acknowledge that this review, like other types of microbiome research, has its limitations, and thus these conclusions should be interpreted within the following contexts: First, the limitations inherent in performing the data analyses, which are detailed above, must be kept in mind. Different methods in the literature might lead to contradictory findings when comparing multiple studies, due to the use of differing experimental designs and technologies. Moreover, the difficulty of generating a comprehensive network of interactions from the existing literature also restricts the conclusions made in this review.

Second, there are also inherent methodological, technical, and biological limitations in the literature that limit our capacity to interpret the significance of *Veillonella* species in the pathogenesis of OSCC. All of the limitations described above for interpreting the data in the paper, as well as the constraints on experimental designs, have significant consequences for our understanding and ability to translate *Veillonella*-related findings into clinically actionable interventions. Overall, the study design should be carefully evaluated to interpret the results accurately, since a correlation does not represent a causation. Also, technical limitations such as small sample sizes, varying patient populations, and differences in oral microbiota due to location, make it difficult to draw a definite conclusion from the published data.

In the future, it would be advantageous to have standardized, high-throughput methodologies to enable us to analyze the multiple interactions in the oral environment and to perform experiments in controlled environments to establish a more complete understanding of their effects. Larger cohorts of individuals and more longitudinal studies are needed to test hypotheses generated by cross-sectional studies. More detailed species- and strain-level analyses of the microbiome are needed to identify subtle ecological relationships between different microbial taxa. Furthermore, functional experimentation and the manipulation of populations and *Veillonella* species can provide insights into how different microbial taxa could directly impact human health in oral carcinogenesis. Finally, there is a vast possibility for applying microbial data to cancer risk analysis and prognosis. Personalized therapies can be directed against tumor-promoting microbes such as *Veillonella* in OSCC as well as to increase tumoricidal or tumor-inhibiting bacteria. In addition, screening of cancer-associated microbes may be useful in determining the degree of surgical removal of the OSCC tumor.

As with all integrative reviews, this paper represented a challenge in balancing synthesis and accuracy because of to the heterogeneity among different studies. It must be acknowledged that some degree of bias may be present. We, however, adopted a transparent approach by explicitly describing our procedures for study inclusion, the development of the interaction network, and the interpretation of conflicting evidence. We hope that this paper makes a modest contribution to a greater appreciation of the potential ecological complexity of the oral environment in disease. This highlights the need to continuously refine research methodologies to accurately address our research goals.
